# Adverse perinatal events, treatment gap, and positive family history linked to the high burden of active convulsive epilepsy in Uganda: A population‐based study

**DOI:** 10.1002/epi4.12048

**Published:** 2017-03-13

**Authors:** Angelina Kakooza‐Mwesige, Donald Ndyomugyenyi, George Pariyo, Stefan Swartling Peterson, Paul Michael Waiswa, Edward Galiwango, Eddie Chengo, Rachael Odhiambo, Derrick Ssewanyana, Christian Bottomley, Anthony K. Ngugi, Charles R. J. C. Newton, the SEEDS Writing Group, Ryan Wagner, Rhian Twine, Myles Connor, F Xavier Gómez‐Olivé, Mark Collinson, Kathleen Kahn, Stephen Tollman, Honorati Masanja, Alexander Mathew, Martin Chabi, Evasius Bauni, Gathoni Kamuyu, Victor Mung'ala Odera, James O Mageto, Ken Ae‐Ngibise, Bright Akpalu, Albert Akpalu, Francis Agbokey, Patrick Adjei, Seth Owusu‐Agyei, Immo Kleinschmidt, Victor C K Doku, ePeter Odermatt, Brian Neville, Josemir W Sander, Stev White, Thomas Nutman, Patricia Wilkins, John Noh

**Affiliations:** ^1^ Makerere University Centre for Health and Population Research Iganga‐Mayuge Health and Demographic Surveillance Site Iganga Uganda; ^2^ Department of Paediatrics and Child Health Makerere University College of Health Sciences Kampala Uganda; ^3^ Astrid Lindgren Children's Hospital Neuropediatric Research Unit Karolinska Institutet Stockholm Sweden; ^4^ Department of International Health Johns Hopkins Bloomberg School of Public Health Baltimore Maryland U.S.A.; ^5^ International Maternal and Child Health Unit Department of Women's and Children's Health Uppsala University Uppsala Sweden; ^6^ Karolinska Institutet Stockholm Sweden; ^7^ UNICEF New York New York U.S.A.; ^8^ Centre for Geographic Medicine Research Coast Kenya Medical Research Institute Kilifi Kenya; ^9^ Department of Infectious Disease Epidemiology Faculty of Epidemiology and Population Health London School of Hygiene and Tropical Medicine London United Kingdom; ^10^ Population Health Sciences Faculty of Health Sciences Aga Khan University (East Africa) Nairobi Kenya; ^11^ Neurosciences Unit UCL Institute of Child Health London United Kingdom; ^12^ Clinical Research Unit London School of Hygiene and Tropical Medicine London United Kingdom; ^13^ Department of Psychiatry University of Oxford Oxford United Kingdom

**Keywords:** Adverse perinatal events, Population study of epilepsy, Risk factors, Treatment gap, Uganda

## Abstract

**Objective:**

To determine the prevalence of active convulsive epilepsy (ACE) and describe the clinical characteristics and associated factors among a rural Ugandan population.

**Methods:**

The entire population in Iganga/Mayuge Health Demographic Surveillance Site (IM‐HDSS) was screened using two questions about seizures during a door‐to‐door census exercise. Those who screened positive were assessed by a clinician to confirm diagnosis of epilepsy. A case control study with the patients diagnosed with ACE as the cases and age/sex‐matched controls in a ratio of 1:1 was conducted.

**Results:**

A total of 64,172 (92.8%) IM‐HDSS residents, with a median age of 15.0 years (interquartile range [IQR]: 8.0–29.0), were screened for epilepsy. There were 152 confirmed ACE cases, with a prevalence of 10.3/1,000 (95% confidence interval [CI]: 9.5–11.1) adjusted for nonresponse and screening sensitivity. Prevalence declined with age, with the highest prevalence in the 0–5 years age group. In an analysis of n = 241 that included cases not identified in the survey, nearly 70% were unaware of their diagnosis. Seizures were mostly of focal onset in 193 (80%), with poor electroencephalogram (EEG) agreement with seizure semiology. Antiepileptic drug use was rare, noted in 21.2% (95% CI: 16.5–25.8), and 119 (49.3%) reported using traditional medicines. History of an abnormal antenatal period (adjusted odds ratio [aOR] 10.28; 95%CI 1.26–83.45; p = 0.029) and difficulties in feeding, crying, breathing in the perinatal period (aOR 10.07; 95%CI 1.24–81.97; p = 0.031) were associated with ACE in children. In adults a family history of epilepsy (aOR 4.38 95%CI 1.77–10.81; p = 0.001) was the only factor associated with ACE.

**Significance:**

There is a considerable burden of epilepsy, low awareness, and a large treatment gap in this population of rural sub‐Saharan Africa. The identification of adverse perinatal events as a risk factor for developing epilepsy in children suggests that epilepsy burden may be decreased by improving obstetric and postnatal care.

**Key Points**

- Prevalence of epilepsy in Uganda is high and declines with age, with the highest burden seen in children younger than the age of 5 years
- Most seizures are of the focal onset type, with discordance between the seizure semiology and EEG features
- There is low awareness, a large treatment gap, and considerable stigma regarding epilepsy in this population
- Perinatal infections, neonatal insults, and family history are important risk factors associated in the development of epilepsy
- Efforts to improve the antenatal and postnatal care may reduce the overall burden of epilepsy

Epilepsy ranks as one of the most common chronic neurological disorders with significant personal and social impact. Approximately 70 million people are living with epilepsy worldwide^1^ with the majority (>80%) living in low‐ and middle‐income countries (LMICs).^2^ The sub‐Saharan Africa (SSA) region alone is estimated to have at least 10 million persons with epilepsy (PWEs).^3^

There is a paucity of epidemiologic studies designed to determine the prevalence of epilepsy in Uganda and factors associated with the disease. Furthermore, the relatively efficient and inexpensive hospital and medical records–based approaches frequently used in the developed world to determine prevalence are not widely available nor well developed in most SSA countries; this approach may be difficult to implement given poor health services utilization by PWEs in LMICs. A recent cross‐sectional community‐based study conducted in rural Uganda in an area of endemic seasonal malaria specifically examined only disabled children for whom epilepsy was the sole or major complaint.^4^ However, this community study by Duggan relied only on those cases with long‐standing recurrent seizures who were able to come for assessment at the government health centers or church halls, and individuals >18 years were excluded.^4^ The prevalence of epilepsy from Duggan's study and the description of the seizure patterns are not comprehensive because the case identification methods used are subject to selection bias.

Earlier studies of epilepsy in Uganda have largely been small, hospital‐based, and descriptive in nature,^5^, ^6^ with none examining the most widely recognized risk factors. Therefore, findings from such studies are not representative because most PWEs rarely access health care facilities for treatment. There are many reasons for the poor uptake of medical services, including poor understanding of the cause of epilepsy; health systems with few skilled staff and limited investigative facilities; lack of drugs; and inability to afford the cost of chronic treatment.^3^, ^7^

Here we present results of an epidemiological study that was part of a large multisite study conducted across five health demographic surveillance systems (HDSSs) in Africa (http://www.indepth-network.org). The overall aim of this study was to determine the prevalence, risk factors, and outcome of epilepsy in five sites across SSA, namely: Ghana, South Africa, Tanzania, Kenya, and Uganda.^8^

This paper provides detailed descriptive analysis of the demographic and clinical characteristics of people with active convulsive epilepsy (ACE) identified during the population‐based survey conducted in Iganga/Mayuge (IM) HDSS site in Uganda. We specifically set out to determine the prevalence of epilepsy and to describe the clinical characteristics and the risk factors in these individuals.

## Methods

### Study area and population

The IM‐HDSS is located in the eastern region of Uganda in the districts of Iganga and Mayuge near the shores of Lake Victoria. It is a defined area approximately 120 km from the country's capital, Kampala. The HDSS area is predominantly rural but partly periurban around the town of Iganga with some trading areas and spans a total area of about 155 km^2^. At the time of the study, the total population was approximately 69,186 people.

The population is homogeneous, with about 80% from a single ethnic group, the Basoga, whose main livelihood is subsistence agriculture. Most of the population under surveillance is relatively young, with approximately 50% aged 15 years and younger.

### Study procedure

The study was conducted over an 8‐month period from February to October 2009 using a previously described three‐stage survey methodology^9^ to identify the epilepsy cases, as described in the following steps.

#### Stage I: Conducting the screening

During the routine census, the Iganga/Mayuge census team conducted door‐to‐door screening of households by interviewing a senior member of the household (usually fathers or mothers) using two questions about seizures or convulsions. The appropriate terminology in the local language for the key words in the screening questions (i.e., seizures/convulsions) was derived from prior qualitative interviews with elders, community health workers, and caregivers in the community and was pretested in communities outside the study area. One question asked whether anyone had fits or had ever been told that they had experienced convulsions/seizures. The second asked about any experience of episodes in which their legs or arms had jerking movements or they had a history of loss of consciousness. Trained census field workers were Ugandans fluent in English and in local languages spoken in the IM‐HDSS area.

### Conducting the two‐step clinical assessments

#### Step one (stage II): Screening of the community with an epilepsy questionnaire

A team of 10 trained Epilepsy Field Workers (EFWs) then interviewed people who screened positive to any one of the two questions used during stage I of the study to establish whether an epilepsy diagnosis was likely. A 10‐item Epilepsy Questionnaire (EQ) with higher specificity for detecting ACE than the screening questions was used for this purpose. The questionnaire was based on information used in international studies^10^, ^11^, ^12^ and previous work done in Kilifi HDSS.^13^ It was translated into the local vernacular (*Lusoga*) by a team of certified professional translators from the Department of Languages at Makerere University.

#### Step two (stage III): Neurological confirmation at the hospital

All those identified by the EQ as potential ACE patients by the EFW were invited to Iganga Referral Hospital to confirm the diagnosis of ACE and to distinguish subtypes of epilepsy by a clinician. A diagnosis of epilepsy was made when there was a history of recurrent (two or more) seizures unprovoked by any immediate identifiable cause.^14^ Active convulsive epilepsy was defined as at least two unprovoked convulsions (seizures with clonic movements) within the last year.

Additional eligible cases that had previously not been identified during the three‐stage method were included in the clinical examination study. These cases were identified following referral by clinic staff, community leaders, and following a population screening sample used to assess the sensitivity of the three‐stage methodology.

During the clinical examination, in‐depth sociodemographic, medical, and birth history was obtained using pretested questionnaires (different questionnaires were used for individuals younger and older than 18 years). All neurological examination findings were recorded on a standardized form developed for this study following which a blood sample was taken. A trained nurse performed an electroencephalogram (EEG) in those with ACE to classify the types of seizures and epilepsy syndrome according to old and new International League Against Epilepsy (ILAE) criteria^15^, ^16^ and to detect any evidence of focal lesions. EEG was performed using a 16‐channel digital recording system (Grass Technologies, Warwick, RI, U.S.A.), with electrode placement according to the international 10–20 system. All EEGs were interpreted by Eddie Chengo using a protocol founded on standard methods for EEG reporting and in line with the conventional definitions of the principal EEG features commonly assessed in clinical practice. The seizure classification was independently assessed by two neurologists (CRJCN and AKM) to minimize bias.

To compare the three‐stage survey method of this study with the traditional two‐stage methods used in other studies in LMICs, a sample of 5,000 residents of the IM‐HDSS study area was randomly selected from the census database and interviewed by fieldworkers using the EQ. At the end of the assessments, those patients who required further clinical assessment (potential ACE) were given appointments and facilitated to attend Iganga Referral Hospital.

### The case control study

We conducted a case control study with patients diagnosed with ACE during stage III of the prevalence survey as the cases. These were matched to selected population controls using the age bands 0–5, 6–12, 13–18, 19–28, 29–49, and 50 years and older on a ratio of 1:1, randomly chosen from the census database. All controls were invited to the referral hospital for stage III assessment by interviewers and clinicians who were blind to their case‐control status. If any subject recruited as a control was subsequently found to have ACE that subject was reclassified as a case and a suitable replacement was selected from the database.

### Serological testing

Serological tests were conducted on blood samples from cases and controls to determine exposure to neurocysticercosis using a Western blotting technique,^17^ onchocerciasis using a modified ELISA test,^18^ toxocariasis using an ELISA IgG and IgG4 *Toxocara* commercial kit (Cypress Diagnostics, Belgium), toxoplasmosis using the Toxoplasma IgG Elisa kit (Abbott Toxo IgG ELISA, Genesis Diagnostics), and HIV using the ABOTT DETERMINE test (Abbott Park, IL, U.S.A.). 19

### Investigated risk factors

Data on sociodemographic variables, past medical history (history of diabetes mellitus, maternal seizures, or hypertension in the family), family history of seizures, nonclinical associated factors (perinatal events, previous head injuries, diet), and social history (alcohol/recreational drug consumption) were collected by fieldworkers while the clinical history was obtained by clinicians. For the study participants younger than 18 years, the mother or guardian was interviewed and additional questions on antenatal and perinatal events and the presence of pets in the home were included. Adverse perinatal events were defined as difficulties in breathing, crying, or feeding after birth as recalled by the mother. A positive family history of epilepsy was defined as a history of anyone in the family (first‐ or second‐degree relatives) who had seizures (fits). In assessing malnutrition, anthropometric measurements were taken, including body weight, height/length, and presence of edema for children younger than age 5 years. World Health Organization (WHO) Child Growth Standards in monitoring growth and motor development were utilized using the WHO Anthro software^20^ to compute standardized z scores of weight for height in children up to age 5 years, where ≤2 standard deviation (SD) scores indicated malnutrition. For those older than 5 years, body mass index (BMI) was calculated according to the equation: BMI = Weight (Kg)/Height^2^ (m), with a cut‐off of BMI <16 for females and <15 for males indicating malnutrition using WHO standards.

### Statistical analysis

Data were double entered into a mySQL database version 5 open source database (Oracle Corporation, Redwood Shores, CA, U.S.A.) and cleaned by examining outliers and missing values and by comparing the two entries. The data were then transferred into STATA 11 (StataCorp, College Station, TX, U.S.A.) for statistical analysis. The estimated unadjusted prevalence of ACE and 95% confidence interval (CI) was calculated as the number of cases of ACE confirmed in stage III divided by the total population screened in stage I, expressed per 1,000 persons. Multiple imputation^21^ was used to reduce bias in the prevalence estimates due to attrition between stages of the survey. In addition, the prevalence estimates were adjusted for the sensitivity of the three‐stage method.^22^ Logistic regression was used to estimate the odds ratio for the association between each potentially predictive factor and ACE adjusted for confounding resulting from age, sex, and maternal education.

### Ethical approval

Ethical clearance to conduct this study was granted by the School of Public Health Research and Ethics Committee, Makerere University College of Health Sciences, and Uganda National Council for Science and Technology (Ref: HS 663), and by the Committee for the Protection of Human Subjects, University College of London. Written informed consent was sought from each participant in the study.

## Results

### Sociodemographics of the study population screened in Iganga HDSS

From the eligible population of 69,186 persons in Iganga, a total of 64,172 (92.8%) persons were screened for convulsions in the DSS in stage I. The median age of the total population screened in the prevalence part of the study was 15.0 years (interquartile range [IQR]: 8.0–29.0) with similar numbers of females and males (Table 1).

**Table 1:** History, frequency, and occurrence of the seizures

Characteristic/history (n = 241)	Years/N (%)
Median age at onset of the seizures (IQR), in years	6.0 (3.0–13.0)
Family history of seizures	35 (14.5)
Parents with history of seizures	8 (3.3)
Siblings with history of seizures	40 (16.6)
Family history of febrile seizures	48 (19.9)
Seizure frequency (n = 241)	
Daily	33 (13.7)
Weekly	36 (14.9)
Monthly	65 (27.0)
Yearly	101 (42.0)
Uncertain	6 (2.4)
Time of seizure occurrence (n = 241)	
Daytime	41 (17.0)
Nighttime	14 (5.8)
Both day and night time	177 (73.4)
Uncertain	9 (3.7)

IQR, interquartile range.

### Prevalence of epilepsy

**Figure 1:**
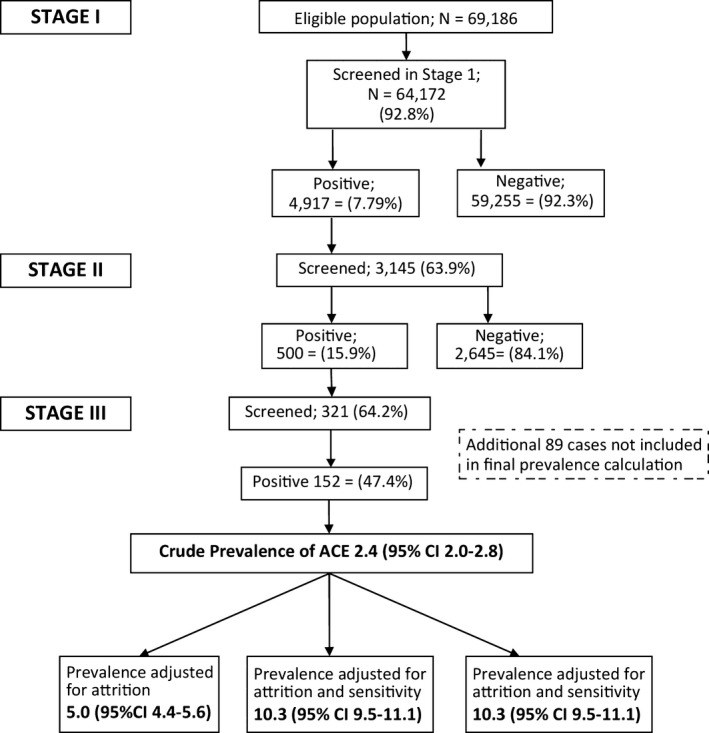
Flow diagram showing selection of study participants and prevalence of active convulsive epilepsy in Iganga‐Mayuge HDSS. ACE‐ active convulsive epilepsy. #On the basis of estimates adjusted for attrition and sensitivity; p = 0.006.

The study procedure and the detailed results of the three‐stage screening survey are provided in Fig. 1.

There were 152 cases with confirmed ACE, giving a crude prevalence rate of 2.4/1,000 (95% CI: 2.1–2.8). The prevalence in males was not significantly higher than in females. After adjusting for attrition and sensitivity of the three‐stage methodology, the epilepsy prevalence was 10.3/1,000 (95% CI: 9.5–11.1).

The median age of these cases was 11.0 (6.0–19.0) years, the median age at onset of their ACE was 2.1 (0.8–6.6) years, and the median duration of their epilepsy was 6.0 (3.0–12.0) years.

#### Age‐specific epilepsy prevalence

**Figure 2:**
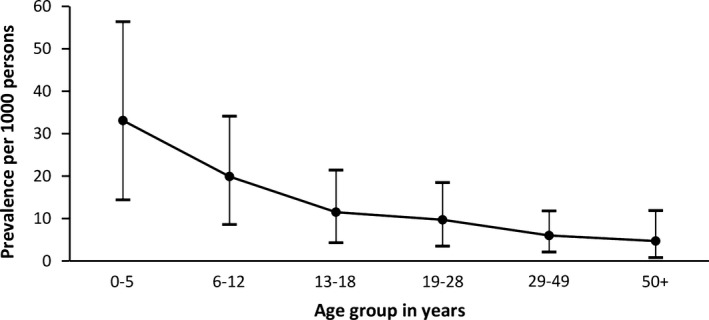
Age‐specific prevalence of active convulsive epilepsy in Iganga‐Mayuge HDSS.

Prevalence varied with age, being highest in the 0–5 years age band and declining with age, as is shown in Fig. 2. The epilepsy prevalence adjusted for age and standardized to the mid‐2000 U.S. population was 6.8 (6.2–7.5) per 1,000 persons.

### Clinical characteristics of the seizures

For this part of the analysis, we used n = 241 cases, which included eligible cases obtained from the population screening sample used to assess the sensitivity of the three‐stage methodology and referrals by the clinic staff and community leaders. Approximately one‐third, or 72 (29.8%) subjects, had ever been told they had fits. There were no differences in sex ratio (males = 121 [50.2%]), and the median age was 10.0 years (IQR: 5.0–19.0).

#### History pertaining to the seizures

The median age at onset of seizures was 6.0 years (IQR: 3.0–13.0). One in 5 cases had a family history of febrile seizures. Other medical history variables are shown in Table 1.

#### Seizure frequency

Seizures occurred daily in only 14% and yearly in 42% (Table 1).

#### Seizure semiology

The most common seizure types were of focal onset (80%), with more than half (55%) being of the motor onset‐clonic type (impaired awareness), as is shown in Table 2.

**Table 2:** Seizure type classifications among n = 241 epilepsy patients

Seizure type^a^	Generalized onset	No. (%)	Focal onset	No. (%)	Unknown onset	No. (%)
	Motor—all with impaired awareness		Focal (clonic) to bilateral tonic‐clonic seizure with impaired awareness	55 (22.8)	Unclassified—impaired awareness	31 (12.9)
	Tonic‐clonic	7 (2.9)				
	Myoclonic	2 (0.8)	Motor onset oral‐facial automatisms	1 (0.4)		
	Absences	7 (2.9)	Non–motor onset cognitive—impaired awareness	3 (1.2)		
	Motor‐epileptic spasms	1 (0.4)	Non–motor onset sensory—impaired awareness	2 (0.8)		
			Motor onset‐clonic—impaired awareness	132 (54.8)		
Total		17 (7.04)		193 (80.07)		31 (12.86)

a Using revised ILAE classifications, 2017, seizure types not exclusive.

#### Electroencephalographic features

The EEG recordings were available for 234 persons with ACE, and of these more than half (129, or 55.1%) were normal, while 102 (43.6%) were abnormal and 3 (1.3%) undetermined. Epileptic activity such as spikes, sharp waves, spike+wave, and rhythmic runs was present in 67/234 (28.6%) of the EEG readings. There was discordance in the seizure semiology and EEG features recorded, with the most frequent abnormal epileptiform activity observed in order of frequency as focal extratemporal 51 (50.0%), focal temporal 48 (47.0%), multifocal 28 (27.5%), and generalized abnormalities 16 (15.7%).

#### Antiepileptic drug use

There was minimal and irregular supply of antiepileptic drugs (AEDs) at the referral hospital during the time of this study and none available at the lower‐level health units. Of the 241 persons with epilepsy, only 51 (21.2%, 95% CI: 16.5–25.8) reported use of AEDs, whereas nearly half (119, or 49.3%) reported using traditional medicines. The epilepsy treatment gap was 78.8%.

### Risk factors for epilepsy

Risk factors for epilepsy in children and adults are shown in Tables 3 and 4. Children with a history of an abnormal antenatal period or difficulties in feeding, crying, or breathing in the perinatal period were 10 times more likely to have epilepsy (adjusted odds ratio [aOR] 10.28; 95% CI: 1.26–83.45) and (aOR 10.07; 95% CI: 1.24–81.97), respectively. In addition, a positive history of seizures in the family (aOR 2.64; 95% CI: 1.21–5.77; p = 0.015) and exposure to *Onchocerca volvulus* (aOR 4.03; 95% CI: 1.16–14.00; p = 0.028) were also associated with epilepsy in the children. Other risk factors are shown in Table 3.

**Table 3:** Risk factors for epilepsy among children (aged < 18 years) in Iganga/Mayuge HDSS

	Children with no ACE	Children with ACE	Unadjusted		Adjusted (age, sex, maternal education)	
	% (n/N)	% (n/N)	OR (95% CI)	p value	OR (95% CI)	p value
Seizures in the family	9.4 (16/171)	24.2 (38/157)	3.09 (1.64,5.81)	<0.001	2.64 (1.21,5.77)	0.015
Maternal seizures	1.2 (2/171)	2.6 (4/155)	2.24 (0.40,12.39)	0.356	1.13 (0.14,8.49)	0.907
Abnormal delivery	1.8 (3/166)	3.2 (5/157)	1.79 (0.42, 7.61)	0.432	0.95 (0.19,4.90)	0.957
Abnormal antenatal period	1.2 (2/167)	8.9 (14/156)	8.13 (1.82,36.39)	0.006	10.28 (1.26,83.45)	0.029
Home delivery	14.1 (24/170)	28.7 (45/157)	2.78 (1.53,5.04)	0.001	3.02 (1.38,6.60)	0.006
Problems after birth	2.4 (4/169)	6.4 (10/156)	2.82 (0.87,9.20)	0.085	3.18 (0.62,16.36)	0.166
Difficulties feeding, crying, or breathing	1.8 (3/166)	5.9 (9/152)	3.42 (0.91,12.88)	0.069	10.07 (1.24,81.97)	0.031
Head injury	0.6 (1/170)	3.9 (6/155)	6.85 (0.81,57.56)	0.076	6.76 (0.79,57.70)	0.081
Malnourished	42.9 (55/128)	38.9 (44/113)	0.85 (0.50,1.42)	0.526	0.86 (0.46,1.63)	0.647
Eats cassava	89.5 (154/172)	94.3 (148/157)	1.92 (0.84,4.41)	0.123	2.03 (0.78,5.29)	0.145
Dogs in household	15.7 (27/172)	6.4 (10/157)	0.36 (0.17,0.78)	0.009	0.44 (0.17,1.14)	0.091
Cats in household	4.1 (7/172)	1.3 (2/157)	0.30 (0.06,1.49)	0.141	0.32 (0.03,3.13)	0.327
Eats pork	10.5 (18/171)	12.8 (20/156)	1.26 (0.64,2.48)	0.505	1.59 (0.68,3.76)	0.285
Malaria IgG + ve (schizont)	99.2 (129/130)	100 (87/87)	1	NA	NA	NA
Hospitalized with malaria or fever	5.8 (10/172)	4.5 (7/157)	0.76 (0.28,2.04)	0.580	0.94 (0.32,2.72)	0.903
*Toxocara canis* IgG4 + ve	18.5 (24/130)	19.5 (17/87)	1.07 (0.53,2.14)	0.842	1.00 (0.39,2.58)	0.998
*Toxoplasma gondii* IgG + ve	20.0 (26/130)	22.9 (20/87)	1.19 (0.62,2.31)	0.598	1.37 (0.57,3.26)	0.479
*Taenia solium* + ve	1.5 (2/130)	0.0 (0/87)	NA	NA	NA	NA
*Onchocerca volvulus* + ve	4.6 (6/130)	12.6 (11/87)	2.99 (1.06,8.42)	0.038	4.03 (1.16,14.00)	0.028
HIV + ve	2.3 (3/130)	3.4 (3/87)	1.51 (0.29,7.66)	0.618	1.66 (0.22,12.46)	0.623

ACE, active convulsive epilepsy; CI, confidence interval; IgG, immunoglobulin G; OR, odds ratio.

**Table 4:** Risk factors for epilepsy among adults (aged ≥ 18 years) in Iganga/Mayuge HDSS

	Adults with no ACE	Adults with ACE	Unadjusted		Adjusted (age, sex, maternal education)	
	% (n/N)	% (n/N)	OR (95% CI)	p value	OR (95% CI)	p value
Seizures in the family	10.8 (9/83)	33.3 (19/57)	4.11 (1.69,9.95)	0.002	4.38 (1.77,10.81)	0.001
Maternal seizures	0.0 (0/84)	1.7 (1/57)	1	NA	NA	NA
Abnormal delivery	2.4 (2/83)	1.8 (1/56)	0.74 (0.06,8.32)	0.80	0.79 (0.07,9.17)	0.852
Home delivery	43.3 (42/97)	35.9 (23/64)	0.71 (0.36,1.41)	0.332	0.59 (0.28,1.23)	0.160
Problems after birth	0.0 (0/79)	7.8 (4/51)	1	NA	NA	NA
Head injury	1.2 (1/81)	8.8 (5/57)	7.84 (0.89,69.07)	0.064	8.12 (0.90, 73.19)	0.06
Drinks alcohol	11.4 (8/70)	16.0 (8/50)	1.48 (0.50,4.14)	0.469	1.29 (0.40,4.13)	0.666
Eats cassava	92.8 (77/83)	100.0 (57/57)	1	NA	NA	
Eats pork	12.9 (9/70)	21.1 (11/52)	1.82 (0.69,4.78)	0.225	1.79 (0.66,4.84)	0.248
Uses drugs	25.3 (21/83)	20.7 (12/58)	0.77 (0.34,1.72)	0.525	0.79 (0.35,1.81)	0.591
Hypertension	1.2 (1/83)	1.7 (1/58)	1.44 (0.09,23.48)	0.799	1.99 (0.10,39.49)	0.650
Stroke	0.0 (0/83)	1.7 (1/58)	1	NA	NA	NA
Diabetes mellitus	0.0 (0/83)	0.0 (0/58)	NA	NA	NA	NA
Malnourished	16.7 (14/84)	24.6 (14/57)	1.63 (0.71,3.74)	0.251	1.54 (0.65,3.60)	0.321
Dogs in household	13.4 (11/82)	10.3 (6/58)	0.74 (0.26,2.14)	0.585	0.75 (0.26,2.19)	0.606
Cats in household	2.4 (2/82)	1.7 (1/58)	0.70 (0.06,7.93)	0.775	0.92 (0.08,10.66)	0.948
Malaria IgG + ve (schizont)	100.0 (73/73)	100.0 (38/38)	NA	NA	NA	NA
Hospitalized with malaria/fever	4.0 (4/100)	7.7 (5/65)	2.00 (0.52,7.74)	0.316	2.28 (0.57,9.14)	0.244
*Toxocara canis* IgG4 + ve	35.6 (26/73)	44.7 (17/38)	1.46 (0.66,3.25)	0.350	1.37 (0.60,3.10)	0.450
*Toxoplasma gondii* IgG + ve	39.7 (29/73)	36.8 (14/38)	0.88 (0.39,1.99)	0.767	0.86 (0.38,1.97)	0.728
*Taenia solium* + ve	0.0 (0/73)	0.0 (0/38)	NA	NA	NA	NA
*Onchocerca volvulus* + ve	8.2 (6/73)	5.3 (2/38)	0.62 (0.11,3.23)	0.571	0.63 (0.12,3.44)	0.599
HIV + ve	2.7 (2/73)	5.3 (2/38)	1.97 (0.27,14.58)	0.506	1.25 (0.15,10.44)	0.834

ACE, active convulsive epilepsy; CI, confidence interval; IgG, immunoglobulin G; OR, odds ratio.

In contrast, in adults (Table 4), the only factor associated with epilepsy was a positive history of seizures in the family (aOR 4.38 95% CI: 1.77–10.81; p = 0.001).

## Discussion

This study investigated the prevalence, clinical characteristics, and associated factors for epilepsy in a rural Ugandan population. The estimated overall epilepsy prevalence was 10.3 per 1,000. The prevalence declined with age. In children, history of complications in the antenatal period and adverse perinatal events were identified as important risk factors, and a family history of seizures was the factor most strongly associated with epilepsy in adults. Focal onset seizures were the most common seizure type. Awareness of epilepsy among cases was low, and many preferred to use traditional medicines rather than AEDs.

### Prevalence of epilepsy

We found a high prevalence of epilepsy in this rural community; however, this figure may not be comparable to the 12.7/1,000 cases of active epilepsy reported in a meta‐analysis of studies in rural low‐resource settings^1^ or the 13.5/1,000 (95% CI 10.2–17.2) in rural areas of Latin American countries,^23^ given that we were screening for ACE, which represents up to 25–50% of all epilepsies in a population‐based study.^9^, ^24^ It is therefore likely that the epilepsy prevalence we measured in this population is much higher.

The unadjusted prevalence of ACE was lower than that of the other demographic sites in SSA yet similar to that from the Ghanaian HDSS site.^1^ Conversely, following adjustment for the screening tool and attrition in the three‐stage survey, our prevalence was the second highest after that of the Tanzanian HDSS site.

The heterogeneity in prevalence in the different HDSS sites could be ascribed to varied environmental or associated genetic factors in each of these settings. Notably, in IM‐HDSS, once the community realized that clinical care was being provided for the ACE cases, several patients who had previously been “hidden” surfaced and presented as cases to the clinic. This situation was responsible for the rise in the number of ACE cases seen in the subsequent clinical studies. Such a situation highlights the issue of social stigma surrounding epilepsy^25^ and the low community awareness regarding this condition.^26^

In the age‐specific prevalence, we observed that prevalence was highest in the age group <5 years and steadily declined with age. This could likely be due to the reported high rate of birth injury, infection, and head trauma during early childhood noted in poor regions of the world^27^ and high premature mortality and spontaneous remission rates. This is supported by our data, which showed that history of perinatal complications was a key associated factor for the children with epilepsy. We also observed a high percentage of cases with a family history of acute convulsions that could have been contributory, as has been confirmed in a recent study.^28^ The decline of prevalence with age could be a result of decreased survival and has been noted in other rural communities.^9^ There have been high mortality rates reported in patients with epilepsy from rural communities in Africa^29^, ^30^ often as a result of status epilepticus, sudden unexpected death, nonaccidental burn injuries during the epileptic attacks, and drowning. Alternatively, it could be due to spontaneous remission of the epilepsy as the patients grew older. This latter hypothesis requires further study of the natural history of epilepsy in this population.

### Clinical characteristics of the seizures

The most common seizure type was focal onset seizures, comprising more than three‐quarters of the seizures in this group of PWEs. There was low awareness regarding epilepsy among the patients, with two‐thirds being unaware they had a diagnosis of epilepsy. The low awareness regarding epilepsy in the rural communities of many LMICs has previously been reported.^31^, ^32^ This information underlines the need to support programs geared at creating awareness among patients with epilepsy and addressing misconceptions attached to epilepsy. The lack of awareness often makes PWEs delay health seeking and also perpetuates stigmatization, discrimination, and exclusion from society.

Focal onset seizures were common in our population, but more than half of the EEG recordings were normal. In the literature, a normal EEG does not exclude epilepsy because around 10% of patients with epilepsy never show interictal epileptiform discharges (IEDs). About 50% of patients with epilepsy, however, will show IEDs in their first EEG test.^33^ A number of factors may have influenced our EEG findings. The circadian variation may have played a part, especially as has been reported in idiopathic generalized epilepsies. Second, the seizure frequency in our population was low, with more patients reporting seizures yearly rather than daily. Patients with frequent (one per month) seizures have been noted to be more likely to have IEDs than those with rare (one per year) attacks.^34^ Third, the timing of the EEG recording may have been contributory: investigations done within 24 h of a seizure have been shown to reveal IEDs in 51% compared with 34% in those who had a later EEG.^35^ Whereas equipment maintenance in LMIC settings is a recognized challenge, we believe that this issue was unlikely to have had an impact on our EEG findings because precautions were taken to ensure that the EGG equipment was well calibrated, stored under lock and key, and managed by only one trained nurse.

The discordance in the seizure semiology and EEG features seen in our study may indicate localized structural pathology underlying the seizure disorders in our population or diffuse cortical dysfunction as is seen in symptomatic generalized epilepsies.^33^ This explanation may further support the hypothesis of a high rate of infection and traumas during early childhood as a common phenomenon in this population.

### Treatment in epilepsy

This study noted an epilepsy treatment gap of close to 80%, in keeping with the ranges reported for LMICs.^36^, ^37^ The treatment gap is a crucial global challenge of epilepsy care in LMICs. The large treatment gap could have been compounded by the low awareness of epilepsy, the affordability and availability of care and treatment, or likely social stigma surrounding the disease. Furthermore, health system issues such as absence of primary health workers trained to diagnose and treat epilepsy coupled with limited access to the health facilities, especially in rural areas, may have been contributory. It would be important to ascertain the actual causes of not seeking modern health care in this rural community by conducting further studies.

The use of traditional therapy for epilepsy was popular in this community, with nearly a half reporting the use of traditional medicines. Traditional medicine usage is common in SSA and likely stems from the traditional animistic religious beliefs,^38^ which impel PWEs to seek this treatment in preference to biomedical treatment as a result of their perceptions and superstitions about the origins and causes of disease. The use of traditional medicines requires further study to analyze its benefits as well as to consider the possibility of incorporating the traditional healer as an important stakeholder in the management of this complex health condition.

### Risk factors for epilepsy

In general, a history of complications during the perinatal period was associated with epilepsy in this population. Similar findings have been noted in rural studies done within the region.^39^, ^40^ This finding is potentially of public health importance because it points toward the need for ensuring a successful, closely monitored pregnancy period for Ugandan mothers and rigorous emergency and obstetric care for the mother‐baby pair. Preventive measures directed at improvement in these areas may play a role in reducing the risk of epilepsy in the child population.

We noted that epilepsy was associated with onchocerciasis antibody positivity in those younger than 18 years. Whereas the reason for this association still remains unknown, this finding is in agreement with several other studies that have indicated high prevalence of epilepsy in onchocerciasis endemic areas, especially in northern Uganda where the “nodding syndrome” (NS) has been described^41^, ^42^ and in other regions of Africa.^43^

Adults were more likely to have epilepsy if they had a positive family history of seizures. The strong association of family history of seizures with epilepsy for both children and adults has consistently been noted in previous studies done in Africa^9^, ^39^, ^40^ as well as in other population studies,^44^, ^45^ which noted a two‐ to fourfold increased risk of epilepsy among the first‐degree relatives of people with genetic epilepsies or epilepsies whose cause was unknown, compared with the general population.

This finding suggests that genetic factors may play a significant role in the etiology of epilepsy in our population, or these results may denote a higher awareness of seizures and epilepsy in persons who had a positive family history of epilepsy.

### Limitations and strengths

Notably in our study we only detected convulsive epilepsies and heavily relied on the history from the main caregiver and adult patients. For children, it is possible that mothers may be more likely to remember and report a child as having had difficulty, say, with breathing or feeding if such a child was subsequently identified as having a convulsive disease. These narratives may have been prone to recall and response biases, and the clinical classification method that we used may have underestimated the presence of focal onset seizures that rapidly generalize to bilateral tonic clonic. Furthermore, the high nonresponse rate we initially obtained in the first stage of the survey, which we attributed to social stigma, may have affected the actual number of patients identified, though we adjusted for the sensitivity of the screening methodology. The prevalence of febrile seizures in this population was high, and though we used evidence of fever witnessed by the caregiver as a distinguishing feature between febrile and nonfebrile seizures, this classification may not be very precise because determination of the presence of fever in some cases was subjective. The demarcation of actual epileptic seizures from psychogenic nonepileptic seizures (PNESs) and syncope may be difficult by history taking only.

All our EEG recordings were done once, during the day time and for the relatively short duration of a routine EEG. This methodology may have decreased the yield since some patients show discharges mainly in sleep. Higher yields have been reported with repeated as well as prolonged inter‐ictal recording EEG recordings.^33^

Despite our study's methodological limitations, this by far is the largest and most comprehensive community survey of epilepsy in Uganda. Our study findings enhance the knowledge regarding epilepsy epidemiology in Uganda, and the study has several potential strengths. The study site provided an ideal platform to generate population‐based data to inform national health policies and evaluate innovations in assessing the burden of epilepsy in Uganda. Two neurologists independently reviewed each patient's clinical characteristics, and EEG records were interpreted using standard criteria based on the ILAE guidelines.

## Conclusions and Recommendations

There is a significant burden of epilepsy in this rural area of Uganda mostly in children younger than 5 years. The social stigma, irregular AEDs supply, and low community awareness regarding epilepsy may compromise the disease's clinical management and delay treatment seeking, thus contributing to the large treatment gap we observed. Perinatal infections, neonatal insults, and genetic factors are important risk factors for epilepsy. These findings suggest that improvement in emergency obstetric and postnatal care may play a role in the prevention of epilepsy in this community. However, endeavors aimed at further understanding the complex interplay of these factors in the etiology of epilepsy are warranted. In addition, the Ugandan Ministry of Health and concerned stakeholders need to utilize a multipronged approach to raise awareness regarding epilepsy identification and management through education of the individual and community. Means should be devised to strengthen health systems, specifically in rural areas, through training of primary health care workers to manage PWEs and incorporate these services into existing local primary health care programs. Also, the availability of AEDs should be increased at all levels of health care. Research to determine the nature and relevant features of the treatment gap and stigma in epilepsy so as to improve the disease's treatment and control are also recommended.

## Disclosure

The authors have no conflict of interest to declare. We confirm that we have read the Journal's position on issues involved in ethical publication and affirm that this report is consistent with those guidelines.

## Additional Contributors

AKN and CRJCN conceived the study. AKN, CB, AK‐M, and CRJCN designed the study. AKN, AK‐M, DN, RO, EC, and CRJCN set up the study. AK‐M, AKN, DN, GP, SP, PMW, and EG coordinated and monitored the study at Iganga/Mayuge HDSS. RO created the database and monitored data from the Iganga/Mayuge HDSS. AKN and CB primarily did the analysis, with input from DS and CRJCN. AK‐M, AKN, CB, and CRJCN wrote the first draft of the manuscript, and all other authors contributed to the subsequent drafts and reviewed and approved the manuscript for final submission.

## Appendix

Members of The SEEDS writing group are: Agincourt HDSS, South Africa: Ryan Wagner, Rhian Twine, Myles Connor, F Xavier Gómez‐Olivé, Mark Collinson, Kathleen Kahn, Stephen Tollman; Ifakara HDSS, Tanzania: Honorati Masanja, Alexander Mathew (deceased); Iganga‐ Mayuge HDSS, Uganda: Angelina Kakooza‐Mwesige, George Pariyo, Stefan Swartling Peterson, Donald Ndyomugyenyi, Paul Michael Waiswa; Kilifi HDSS, Kenya: Anthony K Ngugi, Rachael Odhiambo, Eddie Chengo, Martin Chabi, Evasius Bauni, Gathoni Kamuyu, Victor Mung'ala Odera (deceased), James O Mageto, Charles R J C Newton; Kintampo HDSS, Ghana: Ken Ae‐Ngibise, Bright Akpalu, Albert Akpalu, Francis Agbokey, Patrick Adjei, Seth Owusu‐Agyei; London School of Hygiene and Tropical Medicine, United Kingdom: Christian Bottomley, Immo Kleinschmidt; King's College London, United Kingdom: Victor C K Doku; Swiss Tropical Institute, Switzerland: ePeter Odermatt; University College London, United Kingdom: Brian Neville, Josemir W Sander, Stev White; National Institutes of Health, U.S.A.: Thomas Nutman; Centers for Disease Control and Prevention, U.S.A.: Patricia Wilkins, John Noh.
